# Predicting 2-year neurodevelopmental outcomes in preterm infants using multimodal structural brain magnetic resonance imaging with local connectivity

**DOI:** 10.1038/s41598-024-58682-8

**Published:** 2024-04-23

**Authors:** Yong Hun Jang, Jusung Ham, Payam Hosseinzadeh Kasani, Hyuna Kim, Joo Young Lee, Gang Yi Lee, Tae Hwan Han, Bung-Nyun Kim, Hyun Ju Lee

**Affiliations:** 1 Department of Translational Medicine, Hanyang University Graduate School of Biomedical Science and Engineering, Seoul, Republic of Korea; 2 Department of Communication Sciences and Disorders, University of Iowa, Iowa City, IA 52242 USA; 3 Department of Pediatrics, Hanyang University Hospital, Hanyang University College of Medicine, 222-1, Wangsimni-ro, Seongdong-gu, Seoul, 04763 Republic of Korea; 4 Division of Neurology, Department of Pediatrics, Hanyang University Hospital, Hanyang University College of Medicine, Seoul, Republic of Korea; 5 Division of Children and Adolescent Psychiatry, Department of Psychiatry, Seoul National University Hospital, Seoul, Republic of Korea; 6 Hanyang Institute of Bioscience and Biotechnology, Hanyang University, Seoul, Republic of Korea

**Keywords:** Brain imaging, Prognostic markers

## Abstract

The neurodevelopmental outcomes of preterm infants can be stratified based on the level of prematurity. We explored brain structural networks in extremely preterm (EP; < 28 weeks of gestation) and very-to-late (V-LP; ≥ 28 and < 37 weeks of gestation) preterm infants at term-equivalent age to predict 2-year neurodevelopmental outcomes. Using MRI and diffusion MRI on 62 EP and 131 V-LP infants, we built a multimodal feature set for volumetric and structural network analysis. We employed linear and nonlinear machine learning models to predict the Bayley Scales of Infant and Toddler Development, Third Edition (BSID-III) scores, assessing predictive accuracy and feature importance. Our findings revealed that models incorporating local connectivity features demonstrated high predictive performance for BSID-III subsets in preterm infants. Specifically, for cognitive scores in preterm (variance explained, 17%) and V-LP infants (variance explained, 17%), and for motor scores in EP infants (variance explained, 15%), models with local connectivity features outperformed others. Additionally, a model using only local connectivity features effectively predicted language scores in preterm infants (variance explained, 15%). This study underscores the value of multimodal feature sets, particularly local connectivity, in predicting neurodevelopmental outcomes, highlighting the utility of machine learning in understanding microstructural changes and their implications for early intervention.

## Introduction

Preterm birth is a major cause of long-term neurodevelopmental disability^1^. Preterm infants at highest risk for neurodevelopmental disorders are those born before 28 weeks of gestational age (GA; extremely preterm [EP]), and the prevalence of mild-to-severe neurodevelopmental disorders at 2 years of age is > 50%^2^. Thereafter, with increasing GA, the prevalence of neurodevelopmental disorders decreases to 10% in very-to-late preterm (V-LP) infants born at 28–36 weeks of GA^3–6^. These prognostic trends have led studies to improve our understanding of neurodevelopmental disorders in EP infants and identify the most vulnerable preterm infants^7,8^. However, increasing evidence of long-term neurodevelopmental delays in V-LP infants, and previous findings suggest that neurodevelopmental outcomes of EP and V-LP differ^9–11^. The developing brain may be affected differently, depending on the level of prematurity^12^. Identifying and understanding the differential factors related to neurodevelopment between EP and V-LP is important for developing early intervention strategies for potentially vulnerable populations^13^.

Studies on neurological underpinnings of preterm birth have shown that the brains of preterm infants are characterized by macro- and micro-structural alterations, such as abnormal white matter (WM) integrity and brain connectivity, as well as morphological changes in the cerebral cortex^14–16^. Neuroimaging studies examining the relationship between magnetic resonance imaging (MRI) indices and neurodevelopment suggest that microstructural abnormalities without brain injury can affect late neurodevelopment^12^. A premature brain is easily exposed to various external stimuli and stress, causing demyelination, axon degeneration, and late migratory neuron reduction, potentially altering the microstructure of the WM^17^. These changes can disrupt the efficient transfer of information between brain regions, thereby affecting overall neurodevelopment^18,19^. Moreover, depending on GA, the normal scheme of myelination in a typical caudorostral pattern may be disrupted^20^. Graph theory-based WM connectome analysis has been used to quantify the efficiency of information transmission in brain regions altered by preterm labor and distinguish between normal and abnormal brain network characteristics^21–23^, demonstrating their relevance to various neurodevelopmental disorders^24–26^.

Recent advances in artificial intelligence have enabled the prediction and interpretation of neurodevelopmental outcomes in preterm infants by modeling complex, nonlinear relationships, and helping clinicians make decisions regarding early intervention and follow-up^27,28^. In classification studies for the identification of high-risk groups for neurodevelopment in preterm infants, a random forest (RF) showed the highest classification accuracy^29^. Another study using logistic regression showed high accuracies of 100 and 88%, respectively, for identifying cognitive and motor delays^30^. However, to provide detailed information on neurodevelopmental severity, prediction of continuous variables may be more effective than that of binary variables^31^. Recent demands in clinical practice emphasize the need for regression models that can predict developmental scores, and studies using convolutional neural network models have been recently conducted^32–34^.

Previous studies predicting neurodevelopmental outcomes in premature infants have used various structural and diffusion MRI measurements as key predictors, along with prenatal, perinatal, and environmental influences^35–37^; however, many of them have limited clinical interpretation because of unimodal or bimodal predictions. Moreover, although the structural connectome contains descriptive information on preterm brain, predicting developmental outcomes using a single predictor may provide incomplete clinical information^38–41^. The predictive utility of multimodality has been demonstrated to improve the predictive accuracy and clinical interpretation of attention deficit hyperactivity disorder^42^ and autism spectrum disorder^43^ classifications. These studies underscore the utility of predictive models that incorporate multiple variable sets, including volumetric, structural network, and clinical variables.

In this study, we applied a machine learning approach to model structural connectivity in the preterm brain. By selecting local connectivity variables through graphical network analysis (GNA) and combining multimodal and multivariate machine learning techniques, we tested the hypothesis that the predictions of structural connectivity change with GA. We aimed to quantify the local connectivity for EP and V-LP groups and identify the variables that contribute to their prediction.

## Results

### Demographics and clinical characteristics

The neonatal intensive care unit neonatal and maternal data, clinical information derived during follow-up, and Bayley Scales of Infant and Toddler Development, Third Edition (BSID-III) subscale results are presented in Table 1. The 193 preterm infants who participated in the study were divided into EP (n = 62) and VLP (n = 131) groups.

**Table 1:** Clinical and maternal characteristics of preterm infants.

	Preterm (n = 193)	EP (n = 62)	V-LP (n = 131)	*p*-value (EP vs. V-LP)
Demographics and maternal characteristics				
Gestational age, mean (SD)	29.87 (3.55)	25.65 (1.4)	31.23 (2.74)	< 0.001
Postmenstrual age, mean (SD)	37.8 (2.16)	38.34 (2)	37.56 (2.38)	< 0.027
Birth weight (g), mean (SD)	1355 (594)	872 (195)	1583 (583)	< 0.001
Small for gestational age, n (%)	28 (15)	9 (15)	19 (14)	1
Male sex, n (%)	85 (44)	28 (45)	57 (44)	0.952
Maternal age, mean (SD)	33.69 (4.13)	33.75 (4.81)	33.66 (3.77)	< 0.896
Maternal education, n (%)				
< 6 years	2 (1)	1 (1.6)	1 (1)	1
< 12 years	37 (19)	13 (21)	24 (18)	0.07
< 16 years	123 (64)	45 (73)	78 (60)	0.003
> 16 years	8 (4)	1 (1.6)	7 (5.3)	0.034
Unknown or not reported	23 (12)	2 (3.2)	21 (16)	N/A
Clinical characteristics				
5 Apgar, mean (SD)	5.1 (1.81)	6.79 (1.63)	6.23 (1.62)	< 0.001
IVH, n (%)	52 (27)	21 (34)	31 (24)	0.187
< IVH grade II, n (%)	7 (13)	4 (19)	3 (18)	0.302
BPD, n (%)	106 (55)	59 (95)	47 (36)	< 0.001
BPD moderate/severe, n (%)	10 (9)	8 (14)	2 (4)	0.003
Follow-up characteristics				
Mean (SD) BSID-III Scores				
Cognitive	99.77 (14.43)	93.92 (13.91)	102.53 (13.83)	< 0.001
Language	93.35 (13.08)	86.82 (13.08)	94.96 (12.23)	< 0.001
Motor	100.6 (15.51)	95 (13.64)	103.25 (15.63)	< 0.001

Data are presented as the mean ± SD or number (%). EP, extremely preterm; V-LP, very-to-late preterm; SD, standard deviation; N/A, not applicable; IVH, interventricular hemorrhage; BPD, bronchopulmonary dysplasia; BSID-III, Bayley Scales of Infant and Toddler Development, Third Edition.

### Local connectivity features with net effect on BSID-III subtests

Table 2 shows GNA results for partial relationship to BSID-III subtests score with WM integrity indices, and factors that directly affect BSID-III scores include positive (+) or negative (−) correlations (Table 2):

**Table 2:** Local connectivity features with partial correlation with each BSID-III subscale score extracted from graphical network analysis.

	Cognitive		Motor		Language	
	Metric	Predictor	Metric	Predictor	Metric	Predictor
1	BC	Rt. ORBsup (+)	BC	Lt. IFGoperc (−)	FA	Rt. PVMT (+)
2		Rt. SFGmed (−)		Rt. IFGoperc (−)	BC	Lt. STG (+)
3		Rt. ORBsupmed (+)		Lt. THA (+)	NL_e_	Rt. ORBsupmed (+)
4		Rt. HIP (+)		Lt. STG (+)		
5		Rt. AMYG (+)		Lt. TPOmid (+)		
6		Lt. LING (+)	DC	Lt. SFGdor (+)		
7		Lt. SOG (+)		Rt. ORBinf (−)		
8		Lt. SPG (+)		Lt. INS (+)		
9	DC	Lt. PreCG (−)		Lt. THA (+)		
10		Rt. ACG (−)		Lt. STG (+)		
11		Lt. PUT (−)		Lt. TPOmid (+)		
12		Rt. PUT (−)	NC_p_	Lt. IFGoperc (+)		
13	NC_p_	Lt. PUT (−)	NL_p_	Lt. INS (−)		
14		Lt. TPOmid (−)		Lt. TPOmid (−)		
15	NL_p_	Lt. PreCG (+)	N_e_	Lt. TPOmid (+)		
16		Lt. DCG (+)		Rt. TPOmid (+)		
17		Lt. PCG (+)	NL_e_	Lt. IFGoperc (+)		
18		Lt. CUN (+)		Lt. CAL (+)		
19		Lt. CAU (+)		Lt. ITG (+)		
20		Lt. PUT (+)				
21		Rt. PUT (+)				
22		Rt. MTG (+)				
23	N_e_	Lt. PCG (−)				
24		Lt. PUT (−)				
25	NL_e_	Rt. PreCG (−)				
26		Lt. PCUN (−)				
27		Lt. PUT (−)				
28		Rt. PUT (−)				
29		Lt. PAL (−)				
30		Rt. PAL (−)				
31		Lt. TPOmid (−)				

Plus and minus symbols indicate positive and negative partial correlations, respectively. For all brain region abbreviations, see table S2 in the supplementary materials. FA, fractional anisotropy; MD, mean diffusivity; RD, radial diffusivity; AD, axial diffusivity; BC, betweenness centrality; DC, degree centrality; NC_p_, nodal clustering coefficient; NL_p_, nodal shortest path length; N_e_, local efficiency; NL_e_, nodal local efficiency; PVMT, white matter pathway connecting primary and secondary visual cortex to middle temporal area.

### Machine learning performance of multimodal feature sets

**Figure 1:**
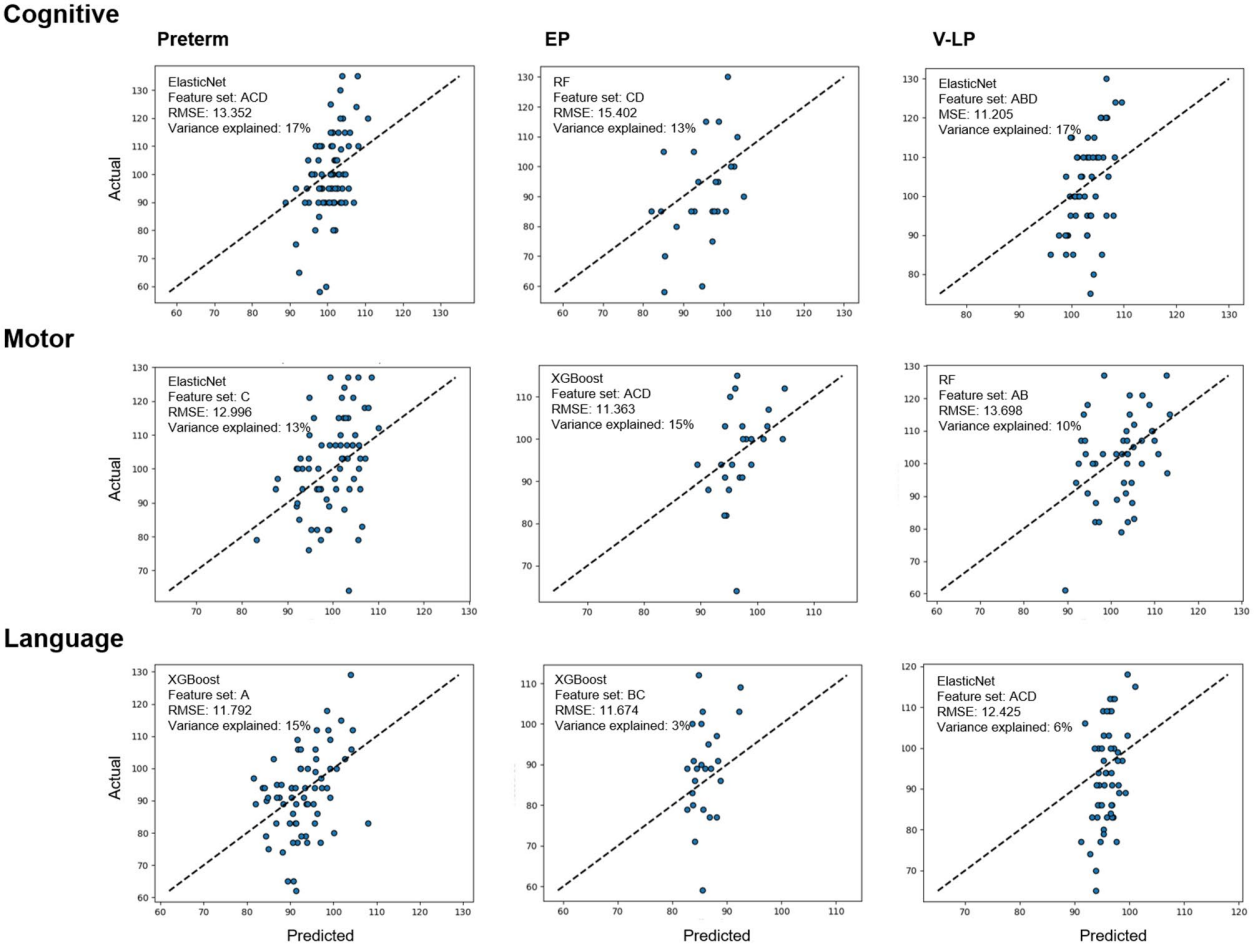
Scatter plot for best predictive model within BSID-III subset. Feature set (**A**) Local connectivity features; (**B**) Clinical characteristic features; (**C**) Volumetric features; (**D**) Global connectivity features. Abbreviations: EP, extremely preterm; V-LP, very-to-late preterm; BSID-III, Bayley Scales of Infant and Toddler Development, Third Edition.

Using multimodal feature sets, we determined the best performing linear and nonlinear models (Table 3; Fig. 1). Regarding the cognitive scores, the preterm (root mean squared error [RMSE], 13.352; variance explained, 17% on ElasticNet) and V-LP (RMSE, 11.205; variance explained, 17% on ElasticNet) groups exhibited the highest predictive performance for models that included local connectivity features. In the EP group, the RF model, including volumetric and global network feature sets, demonstrated the highest predictive performance (RMSE, 15.402; variance explained, 13%). The subgroup that included local connectivity features for motor scores demonstrated the highest predictive performance (EP group: RMSE, 11.363; variance explained, 15% on XGBoost; V-LP group: RMSE, 13.698; variance explained, 10% on RF). Regarding language scores, the preterm group demonstrated a high-performing prediction that included only local connectivity features (Preterm: RMSE, 11.792; variance explained, 15% on XGBoost). However, the other models exhibited relatively low-performance predictions (EP: RMSE, 11.674; variance explained, 3% on XGBoost; V-LP: RMSE, 12.425; variance explained, 6% on ElasticNet).

**Table 3:** Highest predictive models and best performing feature sets.

BSID-III	Group	Top prediction model (predictor sets)	RMSE	Variance explained (%)
Cognitive	Preterm	ElasticNet (Feature set A, C, and D)	13.352	17
	EP	RF (Feature set C and D)	15.402	13
	V-LP	ElasticNet (Feature set A, B, and D)	11.205	17
Motor	Preterm	ElasticNet (Feature set C)	12.996	13
	EP	XGBoost (Feature set A, C, and D)	11.363	15
	V-LP	RF (Feature set A and B)	13.698	10
Language	Preterm	XGBoost (Feature set A)	11.792	15
	EP	XGBoost (Feature set B and C)	11.674	3
	V-LP	ElasticNet (Feature set A, C, and D)	12.425	6

Feature set A: Local connectivity features; B: Clinical characteristic features; C: Volumetric features; D: Global connectivity features.

EP, extremely preterm; V-LP, very-to-late preterm; RF, random forest; BSID-III, Bayley Scales of Infant and Toddler Development, Third Edition; RMSE, root mean squared error.

### Feature importance within the best performing model in each BSID-III subset

**Figure 2:**
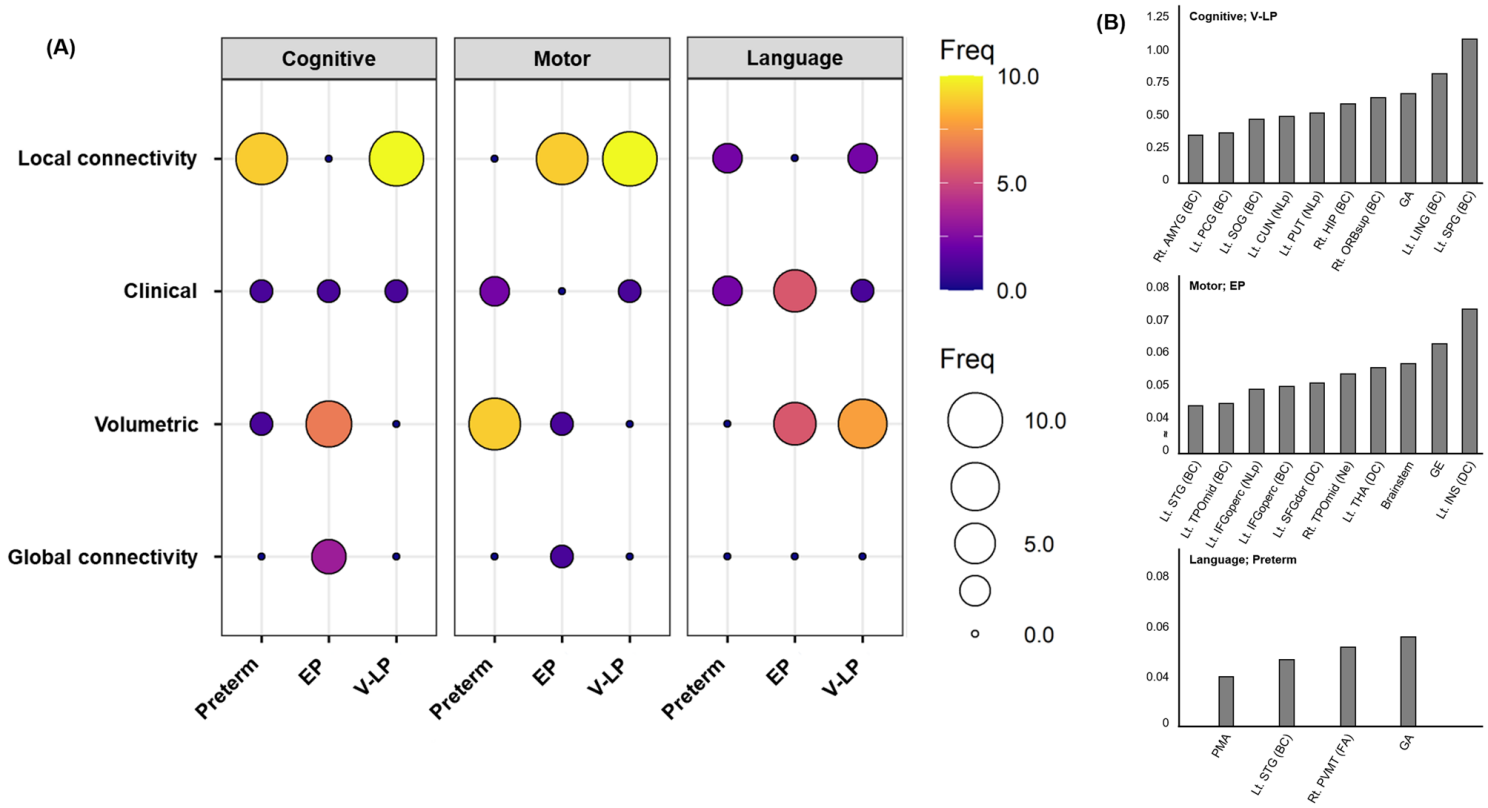
(**A**) Number of importance features in the best performing model in each BSID-III subset frequencies range between 1 and 10. (**B**) Feature importance for the best performing model in all preterm groups. For all brain region abbreviations, see table S2 in the supplementary materials. EP, extremely preterm; V-LP, very-to-late preterm; Freq, Frequencies; BC, betweenness centrality; NL_p_, nodal shortest path length; DC, degree centrality; N_e_, local efficiency; GE, global efficiency; BSID-III, Bayley Scales of Infant and Toddler Development, Third Edition.

The top ten features that were important for prediction were selected, and the quota for each feature set is shown in Fig. 2. Each BSID-III subset showed a different feature importance, depending on the group. In terms of cognitive and language scores, local connectivity and volume predictors had the highest proportions, whereas in motor scores, clinical variables and volume predictors (e.g., cerebellum) had the highest proportions.

**Figure 3:**
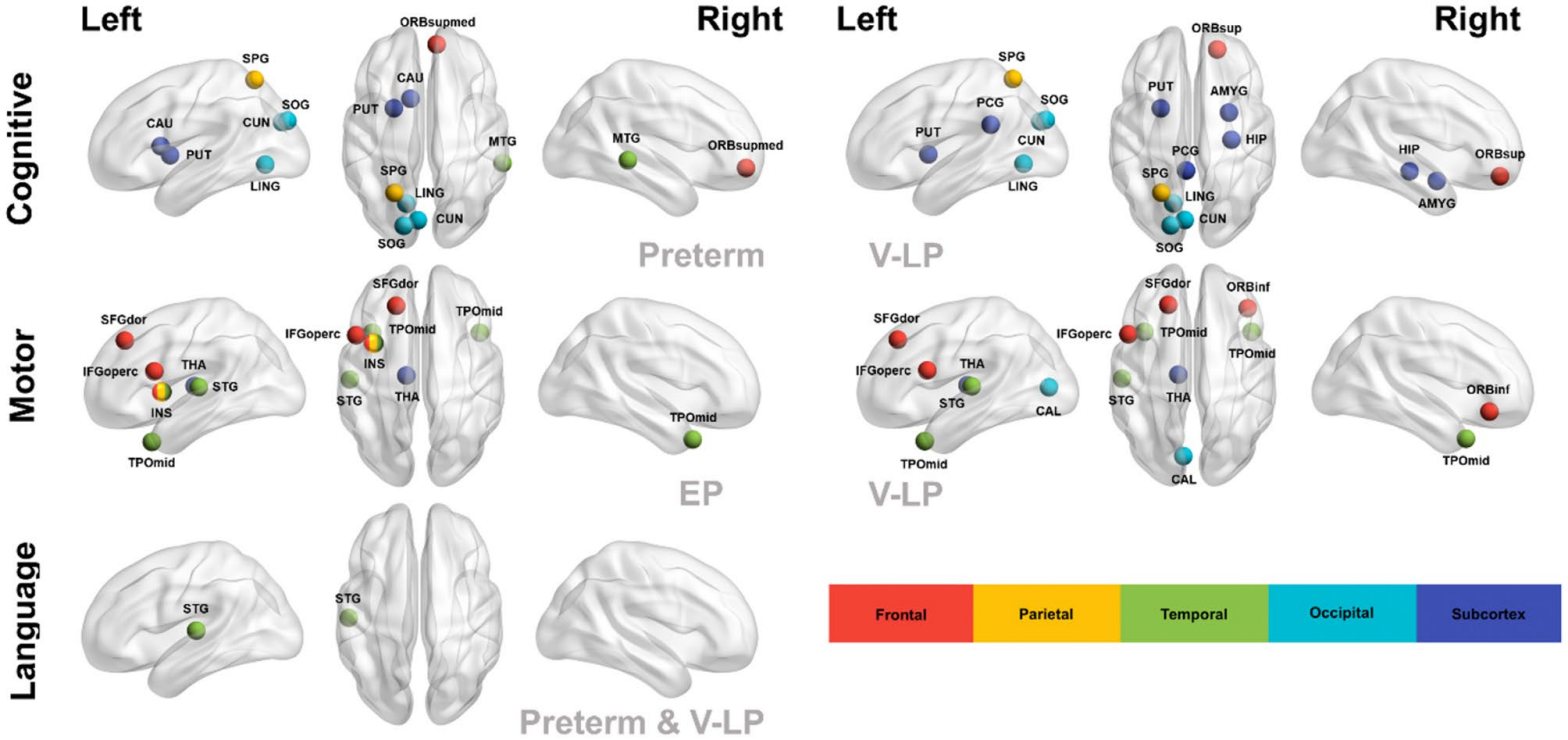
Visualization of the hemispheric distribution of predictors presented by the best performing feature set. Each brain region is represented by a color assigned to its brain lobe. For all brain region abbreviations, see table S2 in the supplementary materials. These images were created using BrainNet Viewer (version 1.7)^44^.

We presented the brain lobe distribution of the local connectivity predictors (Fig. 3) and frequencies of the top 10 predictors from the best-performing models for all BSID-III subsets (Table 4). The feature importance frequency of the brain regions in the local connectivity was ranked by the left superior temporal gyrus (STG), thalamus, and inferior frontal gyrus (opercular), and the remaining regions were counted once or twice.

**Table 4:** Frequencies of feature importance within nine best performing models.

Set	Count	Region (Hemisphere)	BSID-III subset	Group
Local connectivity	4	STG (L)	Language	Preterm and V-LP
			Motor	EP and V-LP
	3	THA (L)	Motor	EP and V-LP
	3	IFGoperc (L)	Motor	EP and V-LP
	2	SOG (L)	Cognitive	Preterm and V-LP
	2	LING (L)	Cognitive	Preterm and V-LP
	2	PUT (L)	Cognitive	Preterm and V-LP
	2	CUN (L)	Cognitive	Preterm and V-LP
	2	SPG (L)	Cognitive	Preterm and V-LP
	2	TPOmid (L)	Motor	EP and V-LP
	2	TPOmid (R)	Motor	EP and V-LP
	2	SFGdor (L)	Motor	EP and V-LP
	2	PVMT (R)	Language	Preterm and V-LP
	1	ORBsupmed (R)	Cognitive	Preterm
	1	CAU (L)	Cognitive	Preterm
	1	MTG (R)	Cognitive	Preterm
	1	ORBsup (R)	Cognitive	V-LP
	1	HIP (R)	Cognitive	V-LP
	1	PCG (L)	Cognitive	V-LP
	1	AMYG (R)	Cognitive	V-LP
	1	INS (L)	Motor	EP
	1	ORBinf (R)	Motor	V-LP
	1	CAL (L)	Motor	V-LP

For all brain region abbreviations, see table S2 in the supplementary materials. EP, extremely preterm; V-LP, very-to-late preterm; BSID-III, Bayley Scales of Infant and Toddler Development, Third Edition.

## Discussion

To the best of our knowledge, the present study is the first to apply linear and nonlinear machine learning methods to predict 2-year neurodevelopmental outcomes in preterm infants, utilizing a comprehensive set of multi-modal features. The predictive performance demonstrated notable improvement when considering multimodal feature sets compared to single-feature sets, with a primary contribution to performance enhancement observed from local connectivity sets. Feature importance in the best-performing models differed, depending on neurodevelopmental subsets, and was primarily ranked in the left STG and thalamus.

Differences in core WM developmental patterns in the EP and V-LP groups suggest that the affected brain regions may differ depending on the degree of prematurity at birth^12^. Preterm groups in this study might not have shared a common etiology, as indicated by differences in WM development patterns, depending on GA. Between 24 and 28 weeks of gestation, thalamocortical afferent axons developed in the frontal, temporal, and occipital areas, and initial synaptic connections and spatial reorganization in the frontal and occipital regions occurred under the influence of sensory-sensitive cortical development^45,46^. In contrast, after 28 weeks of gestation, myelination became prominent, along with astrocyte and oligodendrocyte production, and the sensory-driven development of long-range association fibers of the thalamocortical, somatosensory, visual, and auditory processes occurred^47,48^.

Although the majority of preterm infants performed within the normal range of general cognitive functions^49–52^, previous studies have shown that prediction performance is poor for 2-year-olds with EP who perform at levels 1–2 standard deviation below the expected cognitive function^49,53,54^. Similarly, the significant difference in cognitive scores between EP (93.92 ± 13.91) and V-LP (102.53 ± 13.83) groups may cause performance differences in predicting cognitive outcomes. This suggests that the influence of prematurity level and latent variables early in the EP may cause simultaneous changes in local connectivity and increase collinearity between variables. In this case, global connectivity variables that have been proven in studies comparing pre- and full-term infants at term-equivalent ages may be effective for prediction^21,22,55^.

The emergence of cognitive function is largely influenced by the development of specific subnetworks rather than the development of the whole brain^56^. Differential brain regions identified in this study that predicted cognitive outcomes included the left cuneus, lingual, superior occipital gyrus, and putamen, which are consistent with the regions identified in previous studies^57,58^. They may be responsible for a series of cognitive processes in early brain development, such as the primary processing of visual, and somatosensory information for cognitive^59,60^ and sensory association for higher-order cognitive developments^61^.

Predictors with significant partial correlations with motor outcomes were identified in the thalamus, cerebellum, and frontotemporal regions. The identified predictors shared key biomarkers found in previous neurodevelopmental prediction studies. The thalamus has been identified as an important feature of the preterm subgroup, suggesting that the relationship between the thalamus and motor outcomes may be stratified according to GA^62^. Thalamic development is linearly related to the degree of prematurity^12,63^, which could weaken the thalamocortical connections and lead to the disruption of connections within key brain structures in preterm infants^26,55^. Moreover, a study by Kline et al. showed that the thalamic volume was associated with motor outcomes at 2 years of age^64^, and further correlated with motor function at 7 and 11 years of age^65,66^, suggesting that early thalamic development may have effects that persist throughout childhood and adulthood. Additionally, the feature importance in the left insula and frontal and temporal brain regions may reflect the involvement of the high-level cerebellothalamic pathway in motor development^67^. The left superior frontal gyrus contains part of the premotor cortex and is an important predictor of motor outcomes^33^. Moreover, the insula is partially involved in controlling sustained intentional movements^68^, and the temporal pole is known to play a role in controlling visuomotor movements^69^, suggesting its potential as a key marker for later functional development.

The preterm subgroup exhibited the lowest predictive performance and unreliable feature importance in predicting language scores. These results may be due to the lack of a cohort; therefore, the relationships between multivariate variables and language scores were no longer stratified. Moreover, language performance is more sensitive to potential environmental factors that cannot be completely explained by imaging^70^, and our study may not have considered complex clinical factors derived from the EP group. Nevertheless, the left STG identified in the preterm group was closely related to the language score, again highlighting the importance of this variable as single predictors. A previous study that examined the relationship between language ability at 2 years of age, and local connectivity in preterm infants showed that the left STG was negatively correlated with language scores, suggesting that the left STG is a key region for language development and has microstructural vulnerability^71^.

Although this study attempted to follow the quality assessment criteria provided in recent reviews on predicting pediatric development (Supplementary Table 1)^72^, the most prominent limitation of the current study is the limited amount of data. Generalization to other datasets might be limited due to the lack of external cross-validation. Also, the implementation of a more complex predictive model such as a non-linear SVM or artificial neural network was not possible because of the limited amount of data and poor interpretability of such models. Therefore, the complex relationships between multiple features and predictive value may not be fully represented in the model. A future collaboration between multiple sites can overcome the hurdle of a small sample size. Dependencies among global network metrics exhibiting similar patterns have been identified (Supplementary Fig. 1), which may potentially impact the model's predictive power and should be interpreted with caution^73^. Future research may perform analyses that limit collinearity among metrics while accommodating the inherent biological complexity within each network metric and measuring the uniqueness of network structures^74,75^. Quantification of the structural connectome should be performed with the cerebellum because structural connections within the cerebellum may be important for WM connections around the thalamus. The importance of the local connectivity was primarily identified in the left hemisphere. Given that early brain lateralization is a multivariate trait influenced by a variety of factors, such as stress and the external environment^76–78^. Future studies could utilize lateralization indices in predictive models.

In conclusion, we found that the prediction performance and feature importance differed, depending on the preterm group for the BSID-III. Additionally, the STG and thalamus are important markers for predicting motor and language development. Machine learning approaches that leverage brain connectivity can improve individual risk stratification by improving our understanding of how alterations in the brain microstructure affect neurodevelopment.

## Methods

### Study populations

The participants of the present study included preterm infants born at < 37 weeks GA who were admitted to the neonatal intensive care unit of the Hanyang University Hospital and participated in a follow-up project at the Hanyang Inclusive Clinic for Developmental Disorders between 2017 and 2021. Of 218 eligible preterm infants using the BSID-III^79^, eight preterm infants with brain injury, one with hypothermia, and one with metabolic abnormalities were excluded from image processing. Fifteen of 208 participants were excluded from the WM analysis because of motion artifacts and poor image quality. Similarly, 38 patients were excluded from the volumetric analysis. A total of 193 participants in a WM analysis and 170 in a volumetric analysis were recruited with suitable MRI data obtained at near-term age (postmenstrual age, 35–44 weeks) without congenital brain abnormalities, congenital infections, cystic periventricular leukomalacia, diffuse ventriculomegaly, evidence of genetic disorders, focal abnormalities, intraventricular hemorrhage (IVH, grades II–IV), or punctate WM injury. The Institutional Review Board of the Hanyang University Hospital approved the study protocol, and informed consent was obtained from infant’s parents prior to participation in present study. All procedures were performed in compliance with the principles of the Declaration of Helsinki.

All the preterm infants were assessed at corrected ages between 18 and 24 months by certified examiners for cognitive, motor, and socioemotional development using the BSID-III, with subtests scaled based on age at test. For statistical analysis, preterm groups were divided based on GA 28 weeks (28 < EP; 28 ≥ V-LP).

### Clinical data collection

Detailed information on clinical data was gathered through a systematic and prospective chart review. Clinical variables adopted in this study have useful biological premises and potential associations with later neurodevelopment, and variables that have been validated with improved variance explained in previous papers predicting 2-year neurodevelopmental scores in preterm infants were selected^80^. The present study followed nine predefined clinical factors (GA, postmenstrual age, male sex, maternal education, small gestational age [SGA], IVH, 5-min Apgar score, and bronchopulmonary dysplasia) from the PENUT dataset that are expected to be associated with long-term outcomes^80^. Maternal education level was based on the years of schooling and categorized by educational level.

### Data analysis: Clinical characteristics

The demographics of preterm subgroups were statistically compared using SPSS 27.0 (SPSS. Chicago, IL) software. We utilized the Student’s t-test and chi-square analysis to compare clinical characteristics between preterm subgroups.

### MRI acquisition

Individual preterm infants were scanned at near-term age (postmenstrual age [PMA], 35–44 weeks) using whole-body 3 T magnetic resonance imaging (MRI) scanner (Philips, Achieva, 16-channel phase-array head coil, Best, Netherlands) during natural sleep, using a blanket to preserve body temperature. An experienced pediatrician monitored the pulse oximeter during the MRI to determine the heart and respiratory rates of each infant. Single-shot spin-echo three-dimensional echo planar images were obtained using diffusion tensor imaging (DTI). Parameters of the DTI were b-value = 800 s/mm^2^, echo time = 75 ms, repetition time = 4,800 ms, flip angle = 90°, field of view = 120 × 120 mm, number of electrostatic gradient directions = 32, voxel sizes = 1.56 × 1.56 mm^2^, slice thickness = 2 mm, number of averages = 2, total acquisition time = 6 min 17 s, and water-fat shift = 4.68 Hz/pixel. The slices were axially parallel to the anterior–posterior commissure line with a 40–50 slices covering the entire hemisphere and brainstem. Estimates of motion artifacts of diffusion-weighted images were calculated for individual participants, including absolute and relative volume-to-volume motion and percentage of outliers using the EDDY QC tool^81^ in the FMRIB Software Library (FSL, https://fsl.fmrib.ox.ac.uk/fsl/fslwiki)^82^. Additionally, structural T2-weighted images were acquired for volumetric analysis and to exclude white matter (WM) abnormalities. The parameters for T2-weighted image were echo time = 90 ms, repetition time = 4,800 ms, flip angle = 90°, field of view = 180 × 180 mm^2^, voxel sizes = 0.5 × 0.5 mm^2^, slice thickness = 3 mm, number of averages = 1, total acquisition time = 6 min 30 s, and water-fat shift = 4.68 Hz/pixel.

### Image preprocessing

Imaging data were preprocessed using an eddy correction tool for eddy current distortions, and motion artifacts^83^. A nondiffusion-weighted image (b0 image) was extracted from the raw image, including the skull and nonbrain tissues. To remove the effect of low-frequency intensity inhomogeneity on the b0 diffusion data, the bias field estimated using N4 bias field correction in advanced normalization tools (ANTs)^84^. Subsequently, the principal eigenvalues of the diffusion tensor model were computed by simple least-squares fitting of the diffusion-weighted volume. Fractional anisotropy (FA), mean diffusivity (MD), axial diffusivity (AD), and radial diffusivity (RD) were calculated using tensor-eigenvalues. Quality control of the preprocessed images was performed via visual inspection by two independent reviewers. All procedures were performed using FSL.

### Network construction

A 12-parameter affine transformation and the nonlinear symmetric normalization algorithm from ANTs were employed to transform the individual b0 images into T2-weighted images from the University of North Carolina (UNC) neonate atlas^85^, and vice versa. Inverse transformations were used to warp the automated anatomical labeling atlas from the UNC space to the native space. Discrete labeling values were preserved using nearest-neighbor interpolation, which is a family of sinc-based methods. Using this procedure, we obtained 90 brain regions (each representing a node in the network) for the underlying structural network of each participant (Supplementary Table 2).

Whole-brain fiber tracking using probabilistic tractography was performed for each neonate using FSL. First, to prepare for probabilistic tracking, BEDPOSTX^86^ was used to model the direction of the crossing fiber, and partial volume effects were corrected for thick slices (BEDPOSTX arguments: fiber 3 and Rician for uniform noise levels). Probabilistic tractography was then performed on individual diffusion images using PROBTRACKX^87^. The network matrix, assigned through the connectivity probabilities between brain regions *i* and *j*, was calculated as the total proportion of fibers sampled from all voxels in brain region *i* reaching all voxels in brain region *j* (PROBRACKX arguments are sample tracts per seed voxel: 5,000; step length: 0.5 mm; curvature threshold: 0.2; and fractional anisotropic volumes: 0.01).

Probabilistic tractography depends on the seeding point; therefore, the probability from *i* to *j* can differ from the probability from *j* to *i.* Therefore, we defined the unidirectional connection probability *P*_*ij*_ between regions *i* and *j* and created a 90 × 90 symmetric matrix after averaging these two probabilities. Moreover, we performed pair-wise Pearson correlation for all 4,005 connections with nonzero probability values for all the participants and set *r* = 0.7 as the threshold to remove spurious connections with a small probability of connection. The weighted (*W*) network edges were calculated as *W*_*ij*_ = *P*_*ij*_.

### Global and local network analysis

Prior to brain network quantification, a sparsity threshold of 0.25 (i.e., which is the ratio of the number of actual edges to the maximum possible number of edges in a structural network)^85^, was applied to individual networks to remove the weakest connections subject to experimental noise^88^. The specific threshold selection procedure followed that of our previous network study^23^. Global and local network properties were analyzed using the Brain Connectivity Toolbox^89^ and GRETNA software (http://www.nitrc.org/projects/gretna/)^90^.

Graph metrics were used to quantify brain global (global efficiency, E_glob_; local efficiency, E_loc_; modularity, Q; small-worldness, S; normalized clustering coefficient, C_p_; normalized shortest path length, L_p_)^89^ and local (betweenness centrality, BC; degree centrality, DC; nodal clustering coefficient, NC_p_; nodal shortest path length, NL_p_; nodal efficiency, L_e_; nodal local efficiency, NL_e_)^91,92^ connectivity. Global metrics were computed for 1,000 random networks with conserved number of nodes, number of edges, and degree distribution at predefined sparsity thresholds^23^. Local network metrics were used as indicators of neonatal and children brain development, and employed to elucidate clinical implications^23,93–96^. Details of the described graph-theoretical measures can be found in supplementary text 1.

### WM integrity analysis

We aligned the Johns Hopkins University (JHU) neonatal, probabilistic, WM pathway atlas to the FA images of individual diffusion spaces using a nonlinear symmetric normalization algorithm in the ANTs to compute the mean FA, MD, AD, and RD values for specific WM pathways^97^. This atlas provides 27 major WM pathways in a population-averaged neonatal template.

### Volumetric analysis

To extract neonatal volumetric features, we used the morphologically adaptive neonatal tissue segmentation toolbox (MANTiS) to segment and measure neonatal brain tissue from T2-weighted images^98^. Additionally, MANTiS extends the existing approach to tissue classification implemented in Statistical Parametric Mapping software to neonates, combining template adaptation and topological filtering and segmenting the neonatal brain into eight tissue classes: gray matter, WM, deep gray matter, hippocampus, amygdala, cerebellum, and brainstem. These volumetric values were corrected by dividing them by the total brain volume without cerebrospinal fluid.

### Local connectivity feature

Local connectivity features are inherently complex and high-dimensional and are difficult to describe linearly. This may have caused overfitting because there were more variables than the sample size. GNA is a useful technique for determining conditional effects between a set of observed variables. Additionally, GNA can identify collinear variables and provide estimates of the most transparent relationships among variables through nonlinear relationship modeling. Recently, this methodological approach has provided support for follow-up studies, and has the potential to improve clinical care by identifying variables independently associated with clinical and maternal characteristics and neurodevelopmental outcomes in preterm infants^80^.

We employed GNA to identify the net effect of individual variables on each BSID-III subtest. Four WM integrity indices for 27 Johns Hopkins University pathway atlas and 6 local network properties for 90 brain regions were used to determine whether each of the candidate predictors showed a partial effect, even after considering the clinical characteristics. This analysis uses the method described by Williams and Rast to identify significant correlations with a single variable by forming a matrix of precision for relationships between variables, considering relationships with all other variables^99^. A precision matrix was constructed using the maximum likelihood estimation method, and the Fisher Z-transformation (95% confidence intervals) was performed to establish a network with significant relationships between variables ^99^.

### Linear and nonlinear models: using multimodal feature sets

Four predictor sets were identified. Each predictor group comprised local connectivity features (feature set A), clinical characteristic features (feature set B, n = 12), volumetric features (feature set C, n = 8), and global connectivity features (feature set D, n = 5). The prediction model uses 15 combinations of prediction sets for all possible combinations.

Linear (ElasticNet) and nonlinear regression (RF; XGBoost) analyses for predicting cognitive, language, and motor scores were performed using the presented combination of feature sets. However, GA and postmenstrual age were included in all feature set combinations. All of these regressors were implemented using Python’s scikit learning library (https://github.com/scikit-learn/scikit-learn)^100,101^ except for XGBoost^102^. A randomized hyperparameter optimization was performed with fivefold cross-validation to identify high-performance models with reduced computational costs. The predictive power of the regression models was evaluated by calculating the RMSE on the held-out test set. Randomly selected 30% of the data were used as the held-out test set. We also plotted actual BSID-III scores against predicted scores for all feature set combinations of all the regression models.

### Feature importance

In ElasticNet, feature importance was calculated by penalizing the coefficients in the form of absolute values through the combination of L1 and L2 regulation. In the RF, feature importance was calculated by evaluating the extent to which each feature reduced impurity. Additionally, XGBoost computes feature importance by averaging the information gains from all decision trees into which a particular predictor is split.

### Supplementary Information

Supplementary Information.

## Data Availability

The datasets generated during and/or analyzed during the current study are not publicly available due to inability to share personal information according to research ethics but are available from the corresponding author on reasonable request. Correspondence and requests for materials should be addressed to YHJ (ryanjang93@hanyang.ac.kr) or HJL (blesslee77@hanmail.net).

## Abbreviations

BSID-III: Bayley scales of infant and toddler development, third edition
EP: Extremely preterm
GA: Gestational age
GNA: Graphical network analysis
MRI: Magnetic resonance imaging
RF: Random forest
RMSE: Root mean squared error
STG: Superior temporal gyrus
V-LP: Very-to-late preterm
WM: White matter
