# Supplementary material for: Predicting 2-year neurodevelopmental outcomes in preterm infants using multimodal structural brain magnetic resonance imaging with local connectivity
Source: Sci Rep. 2024 Apr 23;14:9331. doi: 10.1038/s41598-024-58682-8 (PMC11039622; doi:10.1038/s41598-024-58682-8)
Supplement: Supplementary file 1 — Supplementary Information. [file 41598_2024_58682_MOESM1_ESM.docx]

**Supplementary Materials**

**Text S1. Role of network metrics in the altered development of brain networks**

Betweenness centrality has been used as an indicator of communication efficacy and the integration ability of structural and functional connections in neonates ^1^. Furthermore, to elucidate the clinical implications of preterm birth, Lee identified the basis of structural networks in emotional processes following BC lateralization in preterm infants ^2^. Additionally, Fischi-Gomez et al. ^3^ identified the effects of preterm infancy and intrauterine growth restriction on brain development using structural network topologies, such as centrality and clustering coefficients. Moreover, DC can differentiate individuals with typical development from those with autism spectrum disorder (ASD) or attention deficit hyperactivity disorder to support the construction of a network disorder model for disease groups ^4^. Thus, BC and NC_p_ showed significant correlations with brain regions related to the ASD phenotype. In particular, NC_p_ showed a positive correlation between ASD and neurodevelopmental outcomes ^5^. Moreover, Feng et al. ^6^ used BC to differentiate between Alzheimer’s disease and subcortical ischemic vascular dementia.

**Table S1. Quality features for our models**

| **Topic** | **Features to extract** | **Explanation** |
| --- | --- | --- |
| Sample size | Sample size (n) | Sample size was limited to 218 cohorts due to the lack of clinical neonatal brain imaging data. |
| Participants | 1) Clear inclusion and exclusion criteria;  2) Clear description of possible sample attribution | Recommended neonatal and maternal characteristics by Juul et al. 2022. |
| Data leakage | 1) Number of features at start (*n*) and in a final (best-predicting) model (*n*)  2) Feature selection independent of validation performance (Y/N)  3) Number of combinations of hyperparameters tested (*n*, both within and outside the validation or cross-validation) | 1) Start: 565 features; final: 30~40 features  2) Y; Graphical network analysis  3) 30. Hyperparameters were randomly searched within the specified statistical distribution rather than the discrete number of samples. |
| Validation | 1) Internal validation (k-fold, LOOCV, and nested)  2) External validation (Y/N) | 1) k-fold (5~10)  2) N |
| Performance metrics | 1) Outcome type (classification and regression)  2) Base rate (percent abnormal)  3) Type of performance metrics reported (e.g., AUC, accuracy, and sensitivity) | 1) Regression  2) Not applicable  3) RMSE, R2 |
| Interpretability | 1) Interpretability of the model (Y/N)  2) Comparison with previous models (Y/N) | 1) Y; Feature importance from prediction model and correlation analysis between predictors and BSID-III subsets in clinical trials.  2) N |
| Open science | 1) Sharing code, model, or data (Y/N)  2) Decision support model (Y/N) | 1) N; Access to data can be obtained through the corresponding author.  2) N |

N, no; Y, yes.

**Table S2. List of nodes abbreviations (University of North Carolina atlas)**

|  | **Node** | **Abbreviation** |  | **Node** | **Abbreviation** |
| --- | --- | --- | --- | --- | --- |
| 1 | Precentral gyrus left | PreCG.L | 46 | Cuneus right | CUN.R |
| 2 | Precentral gyrus right | PreCG.R | 47 | Lingual gyrus left | LING.L |
| 3 | Superior frontal gyrus (dorsal) left | SFGdor.L | 48 | Lingual gyrus right | LING.R |
| 4 | Superior frontal gyrus (dorsal) right | SFGdor.R | 49 | Superior occipital gyrus left | SOG.L |
| 5 | Orbitofrontal cortex (superior) left | ORBsup.L | 50 | Superior occipital gyrus right | SOG.R |
| 6 | Orbitofrontal cortex (superior) right | ORBsup.R | 51 | Middle occipital gyrus left | MOG.L |
| 7 | Middle frontal gyrus left | MFG.L | 52 | Middle occipital gyrus right | MOG.R |
| 8 | Middle frontal gyrus right | MFG.R | 53 | Inferior occipital gyrus left | IOG.L |
| 9 | Orbitofrontal cortex (middle) left | ORBmid.L | 54 | Inferior occipital gyrus right | IOG.R |
| 10 | Orbitofrontal cortex (middle) right | ORBmid.R | 55 | Fusiform gyrus left | FFG.L |
| 11 | Inferior frontal gyrus (opercular) left | IFGoperc.L | 56 | Fusiform gyrus right | FFG.R |
| 12 | Inferior frontal gyrus (opercular) right | IFGoperc.R | 57 | Postcentral gyrus left | PoCG.L |
| 13 | Inferior frontal gyrus (triangular) left | IFGtriang.L | 58 | Postcentral gyrus right | PoCG.R |
| 14 | Inferior frontal gyrus (triangular) right | IFGtriang.R | 59 | Superior parietal gyrus left | SPG.L |
| 15 | Orbitofrontal cortex (inferior) left | ORBinf.L | 60 | Superior parietal gyrus right | SPG.R |
| 16 | Orbitofrontal cortex (inferior) right | ORBinf.R | 61 | Inferior parietal lobule left | IPL.L |
| 17 | Rolandic operculum left | ROL.L | 62 | Inferior parietal lobule right | IPL.R |
| 18 | Rolandic operculum right | ROL.R | 63 | Supramarginal gyrus left | SMG.L |
| 19 | Supplementary motor area left | SMA.L | 64 | Supramarginal gyrus right | SMG.R |
| 20 | Supplementary motor area right | SMA.R | 65 | Angular gyrus left | ANG.L |
| 21 | Olfactory left | OLF.L | 66 | Angular gyrus right | ANG.R |
| 22 | Olfactory right | OLF.R | 67 | Precuneus left | PCUN.L |
| 23 | Superior frontal gyrus (medial) left | SFGmed.L | 68 | Precuneus right | PCUN.R |
| 24 | Superior frontal gyrus (medial) right | SFGmed.R | 69 | Paracentral lobule left | PCL.L |
| 25 | Orbitofrontal cortex (medial) left | ORBsupmed.L | 70 | Paracentral lobule right | PCL.R |
| 26 | Orbitofrontal cortex (medial) right | ORBsupmed.R | 71 | Caudate left | CAU.L |
| 27 | Rectus gyrus left | REC.L | 72 | Caudate right | CAU.R |
| 28 | Rectus gyrus right | REC.R | 73 | Putamen left | PUT.L |
| 29 | Insula left | INS.L | 74 | Putamen right | PUT.R |
| 30 | Insula right | INS.R | 75 | Pallidum left | PAL.L |
| 31 | Anterior cingulate gyrus left | ACG.L | 76 | Pallidum right | PAL.R |
| 32 | Anterior cingulate gyrus right | ACG.R | 77 | Thalamus left | THA.L |
| 33 | Dorsal (middle) cingulate gyrus left | DCG.L | 78 | Thalamus right | THA.R |
| 34 | Dorsal (middle) cingulate gyrus right | DCG.R | 79 | Heschl gyrus left | HES.L |
| 35 | Posterior cingulate gyrus left | PCG.L | 80 | Heschl gyrus right | HES.R |
| 36 | Dorsal/Middle cingulate gyrus right | PCG.R | 81 | Superior temporal gyrus left | STG.L |
| 37 | Hippocampus left | HIP.L | 82 | Superior temporal gyrus right | STG.R |
| 38 | Hippocampus right | HIP.R | 83 | Temporal pole (superior) left | TPOsup.L |
| 39 | ParaHippocampal gyrus left | PHG.L | 84 | Temporal pole (superior) right | TPOsup.R |
| 40 | ParaHippocampal gyrus right | PHG.R | 85 | Middle temporal gyrus left | MTG.L |
| 41 | Amygdala left | AMYG.L | 86 | Middle temporal gyrus right | MTG.R |
| 42 | Amygdala right | AMYG.R | 87 | Temporal pole (middle) left | TPOmid.L |
| 43 | Calcarine cortex left | CAL.L | 88 | Temporal pole (middle) right | TPOmid.R |
| 44 | Calcarine cortex right | CAL.R | 89 | Inferior temporal gyrus left | ITG.L |
| 45 | Cuneus left | CUN.L | 90 | Inferior temporal gyrus right | ITG.R |

**
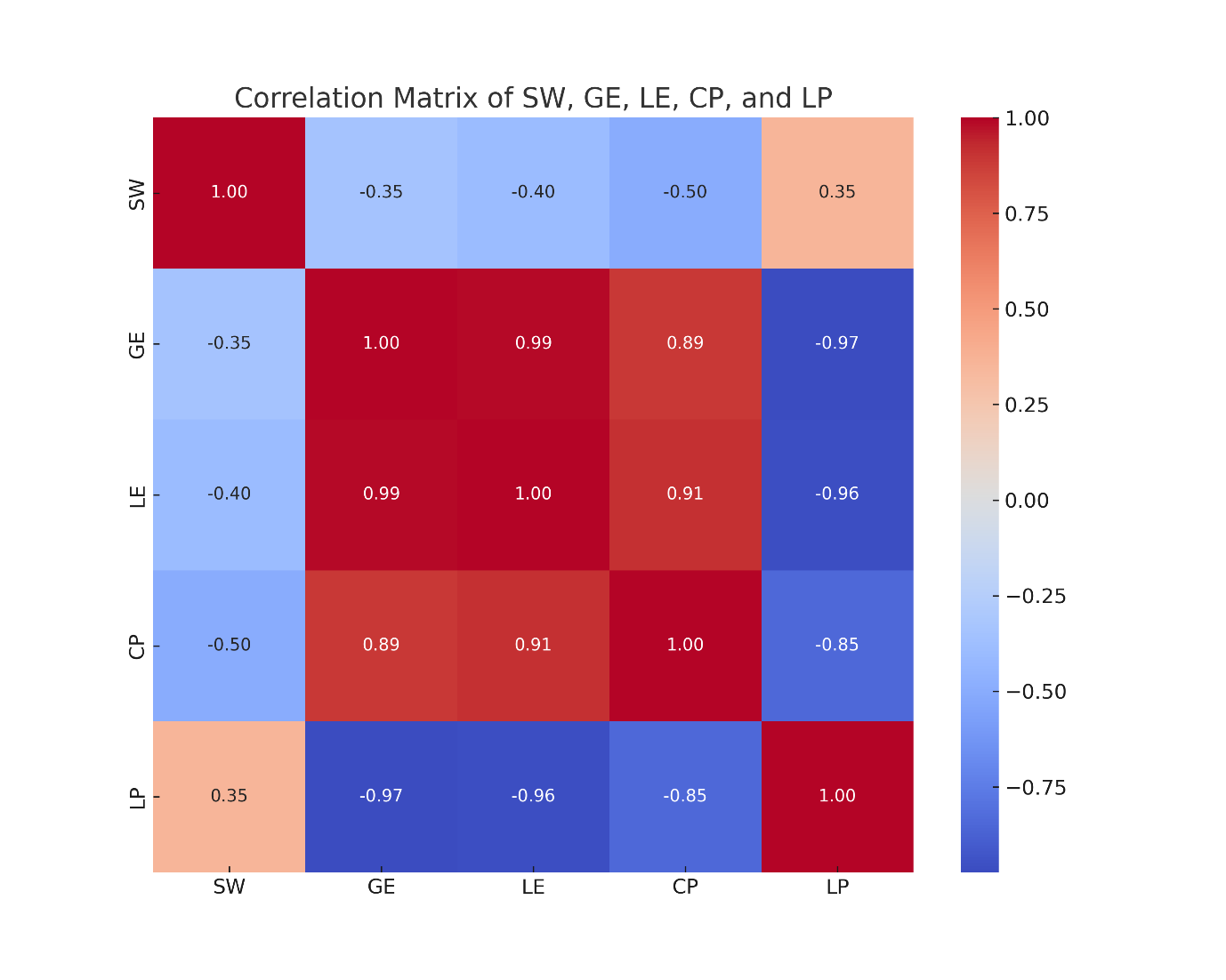
**

**Figure S1.** Correlation matrix of global metrics. Abbreviations: SW, small worldness; GE, global efficiency; LE, local efficiency; CP, clustering coefficient; LP, shortest path length

**References**

1 van den Heuvel, M. P. *et al.* The Neonatal Connectome During Preterm Brain Development. *Cereb Cortex* **25**, 3000-3013, doi:10.1093/cercor/bhu095 (2015).

2 Lee, J. Y. *et al.* Altered asymmetries of the structural networks comprising the fronto-limbic brain circuitry of preterm infants. *Sci Rep* **11**, 1318, doi:10.1038/s41598-020-79446-0 (2021).

3 Fischi-Gomez, E. *et al.* Brain network characterization of high-risk preterm-born school-age children. *Neuroimage Clin* **11**, 195-209, doi:10.1016/j.nicl.2016.02.001 (2016).

4 Di Martino, A. *et al.* Shared and distinct intrinsic functional network centrality in autism and attention-deficit/hyperactivity disorder. *Biological psychiatry* **74**, 623-632 (2013).

5 Billeci, L. *et al.* Brain Network Organization Correlates with Autistic Features in Preschoolers with Autism Spectrum Disorders and in Their Fathers: Preliminary Data from a DWI Analysis. *J Clin Med* **8**, doi:10.3390/jcm8040487 (2019).

6 Feng, M. *et al.* White matter structural network analysis to differentiate Alzheimer’s disease and subcortical ischemic vascular dementia. *Frontiers in Aging Neuroscience* **13**, 650377 (2021).
